# Investigating the impact of long term exposure to chemical agents on the chromosomal radiosensitivity using human lymphoblastoid GM1899A cells

**DOI:** 10.1038/s41598-021-91957-y

**Published:** 2021-06-16

**Authors:** Otilia Nuta, Simon Bouffler, David Lloyd, Elizabeth Ainsbury, Ovnair Sepai, Kai Rothkamm

**Affiliations:** 1 Centre for Radiation, Chemical and Environmental Hazards, Public Health England, Chilton, Didcot, OX11 0RQ Oxon UK; 2 Department of Biology, School of Sciences and Humanities, Nazarbayev University, Kabanbay Batyr 53, 01000 Nur-Sultan, Kazakhstan; 3 Department of Radiotherapy and Radiation Oncology, University Medical Center Hamburg- Eppendorf, 20246 Hamburg, Germany

**Keywords:** Environmental sciences, Risk factors

## Abstract

This study aimed to investigate the impact of chronic low-level exposure to chemical carcinogens with different modes of action on the cellular response to ionising radiation. Human lymphoblastoid GM1899A cells were cultured in the presence of 4-nitroquinoline N-oxide (4NQO), *N*-nitroso-*N*-methylurea (MNU) and hydrogen peroxide (H_2_O_2_) for up to 6 months at the highest non-(geno)toxic concentration identified in pilot experiments. Acute challenge doses of 1 Gy X-rays were given and chromosome damage (dicentrics, acentric fragments, micronuclei, chromatid gaps/breaks) was scored. Chronic exposure to 20 ng/ml 4NQO, 0.25 μg/ml MNU or 10 μM H_2_O_2_ hardly induced dicentrics and did not significantly alter the yield of X-ray-induced dicentrics. Significant levels of acentric fragments were induced by all chemicals, which did not change during long-term exposure. Fragment data in combined treatment samples compared to single treatments were consistent with an additive effect of chemical and radiation exposure. Low level exposure to 4NQO induced micronuclei, the yields of which did not change throughout the 6 month exposure period. As for fragments, micronuclei yields for combined treatments were consistent with an additive effect of chemical and radiation. These results suggest that cellular radiation responses are not affected by long-term low-level chemical exposure.

## Introduction

A number of bodies with interests in radiation protection have highlighted the need to explore the effects of combined exposures to radiation and other agents. These include MELODI^1^ and UNSCEAR^2^.

The scientific literature on the underlying mechanisms associated with the toxicity and carcinogenicity of man-made or naturally occurring chemicals is extensive and it has become very important to compare the findings of different in vitro studies and relate them to risk assessment in humans. For several chemicals, their toxicity is well characterised at high doses, while at low doses their long-term effects and impact on human health are not well known.

In genotoxicity studies, chemical agent-induced effects vary depending on cell type, concentration and duration of exposure and its chemical form. Genetic effects include the induction of chromosome damage^3–8^, DNA strand breaks^9,10^ and DNA protein cross links^11–17^. The mode of interaction with DNA may be direct, by the formation of small or bulky DNA adducts as well as strand breaks, or indirect, by the formation of radicals in the vicinity of DNA, leading to strand breaks or small adducts.

Many studies addressed the issue of the effects of acute combined exposures particularly in cancer therapy. Yet, occupational exposures to chemical agents are generally chronic exposures to low levels and large populations may be exposed^2^. The problem of combined radiation and chemical chronic exposures has received insufficient attention. The most relevant review identified a significant knowledge gap in that *‘…. essentially no guidance has been provided for conducting risk assessment for two agents with different mechanisms of action (i.e. energy deposition from ionising radiation versus DNA interactions with chemicals) but similar biological endpoints (i.e. chromosome aberrations, mutations and cancer)’*^18^.

Our study aimed to explore an approach to meeting this research need. The limited range of genotoxicants chosen reflect a variety of mechanisms of interaction with DNA, although, in principle, the approach could be applied to different agent combinations. In particular we assessed the impact of prolonged exposure to chemical genotoxicants on radiation induced chromosomal effects. A model of a genotoxicant exposure scenario was developed that in a simple fashion mimics daily combined exposure situations in which an individual is exposed chronically to a chemical genotoxicant and is then exposed to ionising radiation in a medical or accidental context. This model is not supposed to reflect real-life environmental chemical exposure but exposure to a variety of genotoxicants with different modes of action.

Chemical agents may increase or reduce sensitivity to subsequent radiation exposure. Exposure to cancer chemotherapeutics including alkylating agents causes widespread changes in gene expression^19^. Deregulated genes may include those involved in DNA double strand break (DSB) repair^20^ providing a possible mechanistic link to the alteration of sensitivity to ionising radiation. Gene transcription can be affected by exposure to different chemicals such as arsenic^21^, hydrogen peroxide (H_2_O_2_)^22^ and 4-nitroquinoline-1-oxide (4NQO)^23^. Notably, low dose exposure to sodium arsenite was reported to interact synergistically with UV radiation to induce mutations and alter DNA repair in human lymphoblastoid cells^24^ and additively with low-LET ionising radiation to induce chromosomal damage^25^.

4NQO is manufactured and used as a model in research. The model carcinogen has generally been characterized as “UV-mimetic” with respect to its genotoxic properties. The carcinogenic and mutagenic properties of 4NQO were first reported in 1957^26^. 4NQO induces malignant transformation in hamster and rat cells in vitro^27–29^ and causes cancer in various tissues in mice and rats^30^. 4NQO also produces oxidative damage and DNA single strand breaks (SSB)^30–32^ and generates reactive oxygen species (ROS), such as superoxide radicals or hydrogen peroxide^33,34^. Other studies have shown the appearance of chromosome and chromatid-type aberrations in peripheral lymphocytes^35^*.* Significant increases in chromatid interchanges, chromatid breaks and dicentrics as well as stable and unstable chromosomal aberrations were observed in cells treated with 10^–5^ M 4NQO^35^. Treatment of human lymphocytes in G1 with 10^–6^ M and 5 × 10^–7^ M 4NQO was reported by Preston and Gooch to form chromosome-type aberrations^36^.

Micronuclei may result from mis-segregation of whole chromosomes or chromosome fragments secondary to chromosome breakage and/or damage to the mitotic spindle^37^. They are widely used as a measure of genotoxicity^38^. DNA damage caused by low concentrations of 4NQO assessed by MN induction in human lymphoblastoid TK6 cells was less frequent than in the p53 mutated cell lines AHH-1^8^.

TK6 cells treated with 4NQO for 24 h showed a significant increase in gene mutations at lower concentrations than any of the MN-inducing doses. Hence, 4NQO probably induces predominantly gene mutations, rather than chromosomal damage^39^.

To our knowledge there has been no quantitative analysis on possible interactions of 4NQO with ionising radiation. Moreover, there are no published data on the impact of chronic low level exposure to 4NQO, N-nitroso-N-methylurea (MNU) or H_2_O_2_ on the effects of ionising radiation.

MNU has been classified as a direct-acting alkylating agent and has been used in the past for the laboratory synthesis of diazomethane and as a cancer chemotherapy agent (alone or in combination with cyclophosphamide). It has been shown that MNU exposure of TK6 lymphoblasts for 20 days induced a linear increase of mutation rates for resistance to 6-thioguanine and trifluorothymidine with increasing concentration^40^.

A study by Imaoka evaluated mammary carcinogenesis initiated by combined exposure to various doses of radiation and the chemical carcinogen MNU, using a rat model and molecular biological approaches. The puzzling result of this study was that radiation and MNU act additively in terms of cancer risk but synergistically in terms of cancer initiation. The authors speculate that there may be interactions at different steps that influence the cancer risk. Tumour promotion appears to be a more important rate-limiting step than initiation for rat mammary carcinogenesis^41^.

H_2_O_2_ occurs naturally at low levels, the public is potentially exposed via consumer products. It rapidly decomposes to water and O_2_ in the environment. DNA damage by reactive oxygen species results in a spectrum of DNA lesions including strand breaks. In fact, Olive and Johnston performed an analysis of the pattern of oxidative DNA damage in Chinese hamster cells exposed to different concentrations of H_2_O_2_ for 30 min and measured the relative induction of repairable SSB and potentially lethal DSB, comparing the ratio of either DNA lesions as measure of random damage versus clustered damage. The results suggest that H_2_O_2_ and X-rays produce either type of damage but whereas H_2_O_2_ produces predominantly random SSB, a large proportion of X-rays damage is clustered^42^.

These different mechanisms may suggest the potential for a supra additive effectiveness in combined exposure to H_2_O_2_ and X-rays which needs to be investigated.

Radiation and chemical exposures are factors that can contribute to the incidence of cancer probably caused through DNA damage. People are exposed to a large number of environmental chemicals in different forms of drugs, pesticides, industrial chemicals, food additives, etc. Because populations are also exposed to natural background radiation and X-ray diagnostic procedures, and additional chemical and radiation exposure is received by patients undergoing therapy for the treatment of cancer, it is of great interest to study the interaction between chronic exposures to low levels of chemical genotoxicants and ionising radiation. Here we worked with an in vitro model that allowed us to investigate sequential exposure (1) to chemicals which have a variety of modes of action (2) followed by a radiation exposure.

Moreover, regulatory studies investigating the effects of exposure to genotoxicants are of limited relevance to public health because they were accomplished using high concentrations of chemicals^43^. The levels are not relevant to the in vivo (environmental) scenario in humans^44–52^.

## Results and discussion

### Initial statistical analyses

There was no evidence of departure from normality for any of the endpoints.

### Pilot studies

#### Acute genotoxic effects of chemicals

For the initial identification of suitable chemical concentrations for long-term exposure of lymphoblastoid GM1899A cells, the micronucleus assay was used to determine the highest concentration that does not induce a significant increase of micronuclei after exposure to chemical for 24 h. The yield of micronuclei provided a good estimate of the overall genotoxic effect while the percentage of binucleated cells reflected how many cells passed through mitosis into the cytochalasin B-induced cytokinesis block during the chosen time and thus provided an indirect measure of cell proliferation.

**Figure 1:**
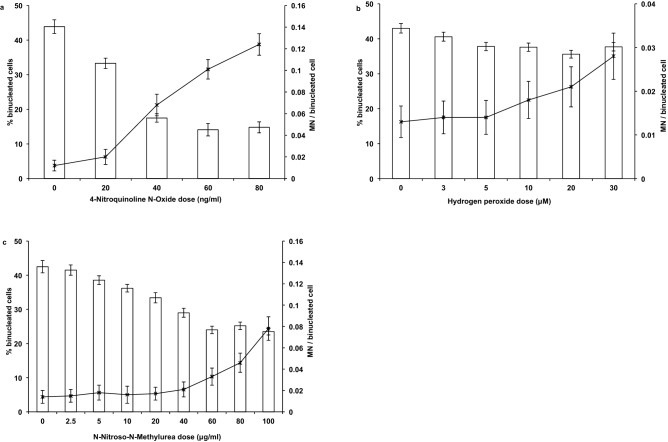
Percentage of binucleated cells (columns) and yield of micronuclei per 100 binucleated cells (‘x’ marker connected by line) following 24 h 4NQO exposure of GM1899A cells (**a**), 24 h H_2_O_2_ exposure of GM1899A cells (**b**), and 24 h MNU exposure of GM1899A cells (**c**). Results are from one experiment, with 200–500 binucleated cells on two slides scored for each dose (**a**) or 500 binucleated cells on two slides scored for each dose (**b**,**c**). Error bars represent Poisson errors.

For 4NQO, Fig. 1a shows a steep reduction in the percentage of binucleated cells (p < 0.001).

Although the effect of chemical was found to be highly significant overall in terms of micronuclei induction (p < 0.001) a concentration of 20 ng/ml did not induce significant levels of micronuclei in binucleated cells (p = 0.060). The concentration of 20 ng/ml (0.06 μM) 4NQO was chosen for long-term exposures.

Hydrogen peroxide induced significant levels of micronuclei (p = 0.001) above a concentration of 5 μM (Tukey’s test for comparison with 0 μM, p = 0.023), with a small but significant effect on cell cycle progression (p = 0.005; Fig. 1b).

MNU treatment reduced the fraction of binucleated cells (p < 0.001) at concentrations that did not induce significant levels of micronuclei (5–40 μg/ml; p = 0.409) (Fig. 1c).

#### Medium term (2 weeks) exposures to chemicals

**Figure 2:**
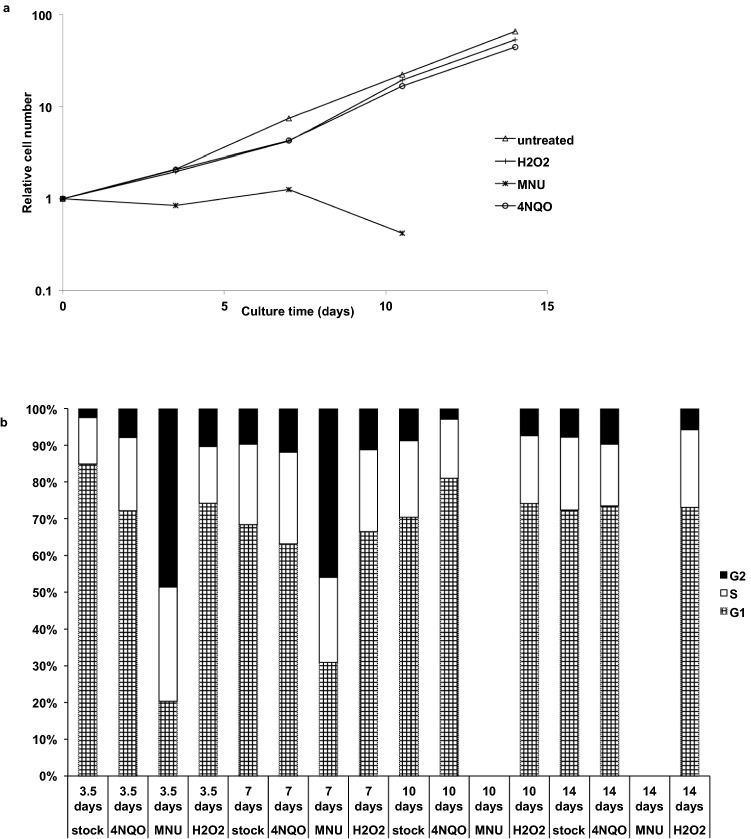
Cell growth (**a**) and flow cytometric analysis of the cell cycle distribution (**b**) of GM1899A cells propagated 1:3 every 3.5 days over a two-week period in the absence (untreated) or presence of 20 ng/ml 4NQO, 10 μM H_2_O_2_, or 20 μg/ml MNU. Relative cell number for each time point was calculated as total cell number for that point divided by total cell number at day 0 and plotted on a logarithmic scale. Cell cycle distributions were determined by propidium iodide-based DNA content analysis. Results are based on one experiment.

**Figure 3:**
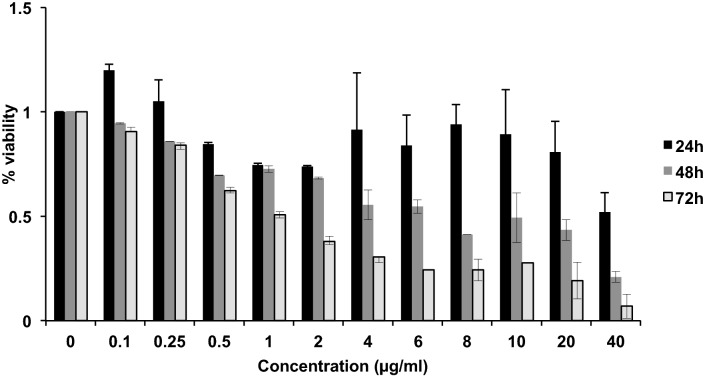
Cell viability analysis of GM1899A cells 24 and 72 h after exposure to different concentrations of MNU. The CellTiter-Blue Cell Viability Assay (Promega) was used. Error bars are standard deviations from 2–3 experiments.

GM1899A cells were initially cultured for two weeks in the presence of 20 ng/ml 4NQO, 10 μM H_2_O_2_ or 20 μg/ml MNU_,_ to confirm whether these concentrations were suitable for long-term exposure experiments. The treatment with 4NQO or H_2_O_2_ was well tolerated over the two week pilot experiment period, causing only a slight (non-significant, ANOVA p for these treatments compared to control = 0.163) reduction in cell production rate (Fig. 2a). During the initial two-week exposure experiment, samples were taken every 3.5 days for flow cytometric analysis of cell cycle distribution changes. Minor, non-significant fluctuations of the cell cycle distribution were recorded both in controls and 4NQO- or H_2_O_2_-exposed cultures (Fig. 2b; p > 0.999). Exposure to 20 μg/ml MNU, however, dramatically reduced the fraction of cells in G1 observed following 3.5 and 7 days of exposure (Fig. 2b). This indicates that cell cycle progression may be blocked in the G2/M phase, indicative of cell cycle arrest and consistent with cell growth data for MNU shown in Fig. 2a. On days 10 and 14 too few cells remained to perform any flow cytometry. O6-methylguanine is considered to be the main toxic lesion induced by methylating agents like MNU and does not seem to induce significant levels of micronuclei at toxic concentrations. However, this lesion induces delayed toxicity which occurs after DNA replication, when repeated unsuccessful attempts to correct a mismatched base opposite the O6-methylguanine base result in the formation of a DNA double-strand break^53^. This delayed effect was observed in cell viability measurements (Fig. 3).

Subsequently, to determine concentrations of MNU that would be compatible with long-term exposure scenarios, cell viability tests were performed for a range of concentrations. Figure 3 shows that even at a concentration of 2 μg/ml, less than 50% of cells were viable three days after the exposure, despite good viability at 24 h. Doses ranging from 0.1 to 1 μg/ml demonstrated a decrease in viability from 95 to 73% with increasing dose at 48 h, but a significantly steeper decrease from 91 to 51% at 72 h. Over the dose ranges assessed the dose of 0.25 μg/ml MNU was well tolerated by the cells at all three time points measured and was therefore subsequently used for long-term exposures.

### Main study: long-term exposures to chemicals

GM1899A cells were chronically exposed to 20 ng/ml 4NQO, 10 μM H_2_O_2_, 0.25 μg/ml MNU or sham-exposed in a set of long-term experiments. Cells were split and medium and chemical renewed every 3.5 days. Samples of cells from each chemical exposure were exposed to 1 Gy of X-rays every four weeks and processed for 1) flow cytometry to monitor changes in cell cycle distribution and apoptosis, 2) chromosome aberration analysis and 3) the micronucleus assay.

**Figure 4:**
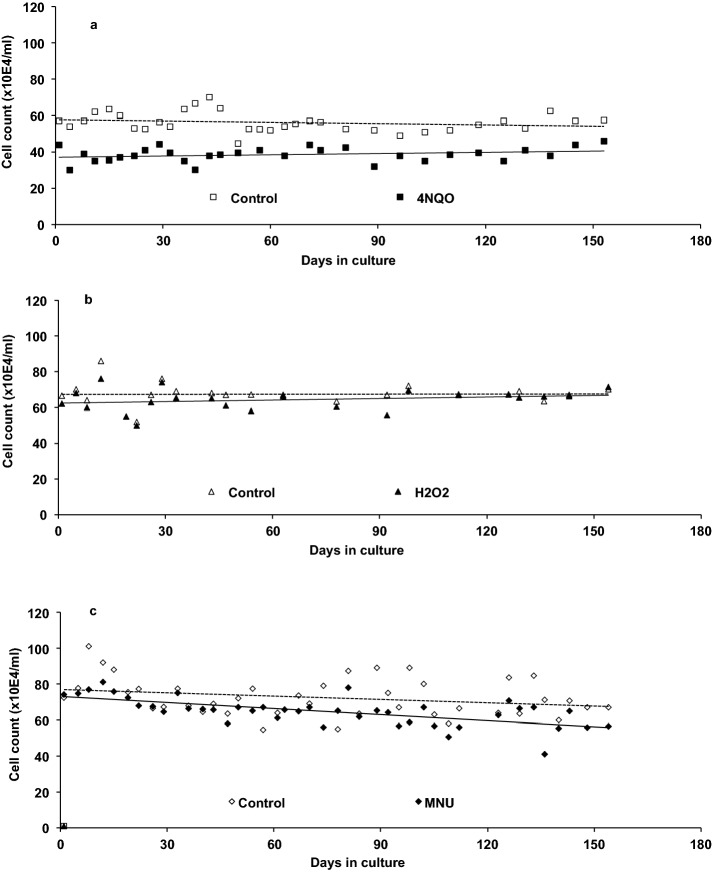
GM1899A cell counts over a six-month period in the absence or presence of 20 ng/ml 4NQO (**a)**, 10 μM H_2_O_2_ (**b**) or 0.25 μg/ml MNU (**c**). Cultures were split every 3.5 days and cell numbers obtained using an automated cell counter. Error bars are standard deviations.

Throughout the 6 months exposure experiments cell counts were obtained. Cell growth results in Fig. 4a show that exposure to 20 ng/ml 4NQO was well tolerated over the 6 months period, with no significant effect of the chemical on cell numbers over time (p = 0.883), although the treatment resulted in a consistent 20% reduction in cell numbers for treated cells over the time period (p < 0.001). Exposure to 10 μM H_2_O_2_ (Fig. 4b) or to 0.25 μg/ml MNU (Fig. 4c) was similarly well tolerated over the 6 months period, though both the chemical and time did induce a small, but significant, reduction in cell numbers during this time (p all < 0.001).

**Figure 5:**
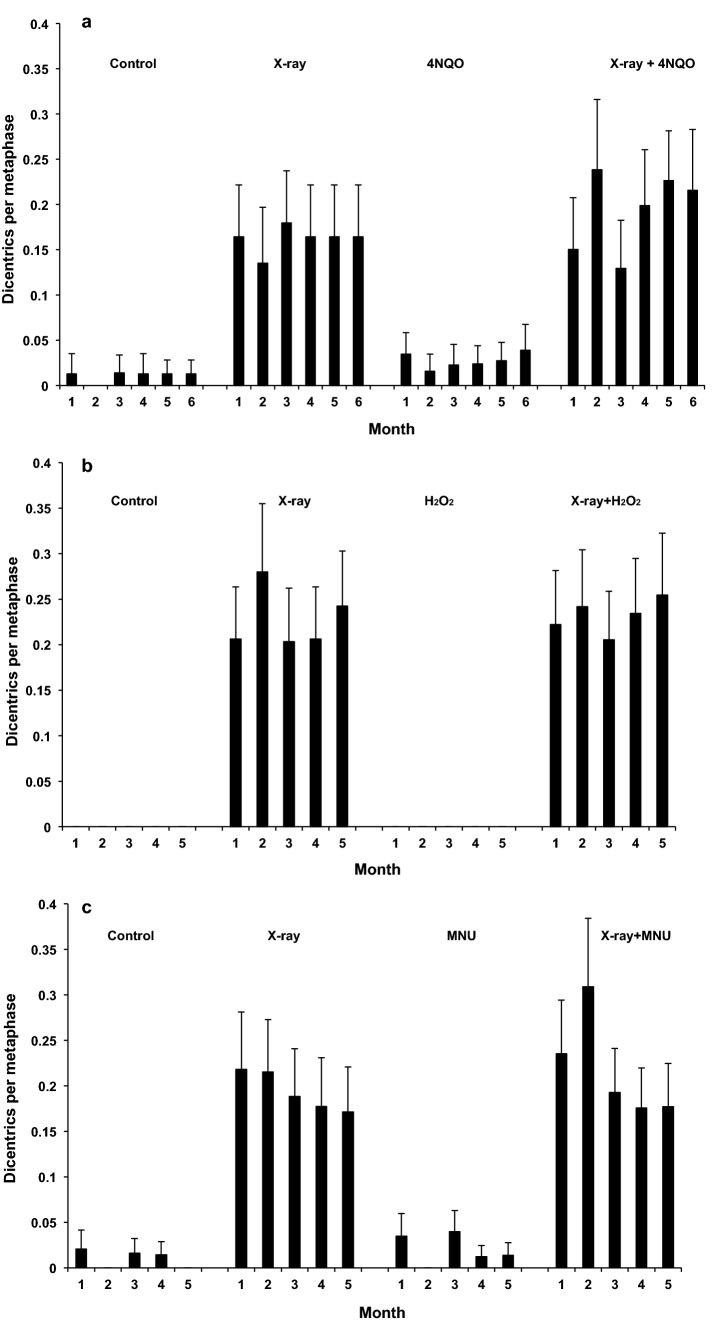
Dicentrics per diploid metaphase after 1–6 months of chronic exposure to: 20 ng/ml 4NQO or sham-exposure and/or acute exposure to 1 Gy X-rays (**a**); 10 μM H_2_O_2_ or sham-exposure and/or acute exposure to 1 Gy X-rays (**b**); 0.25 μg/ml MNU or sham-exposure and/or acute exposure to 1 Gy X-rays (**c**). Error bars are Poisson errors.

**Figure 6:**
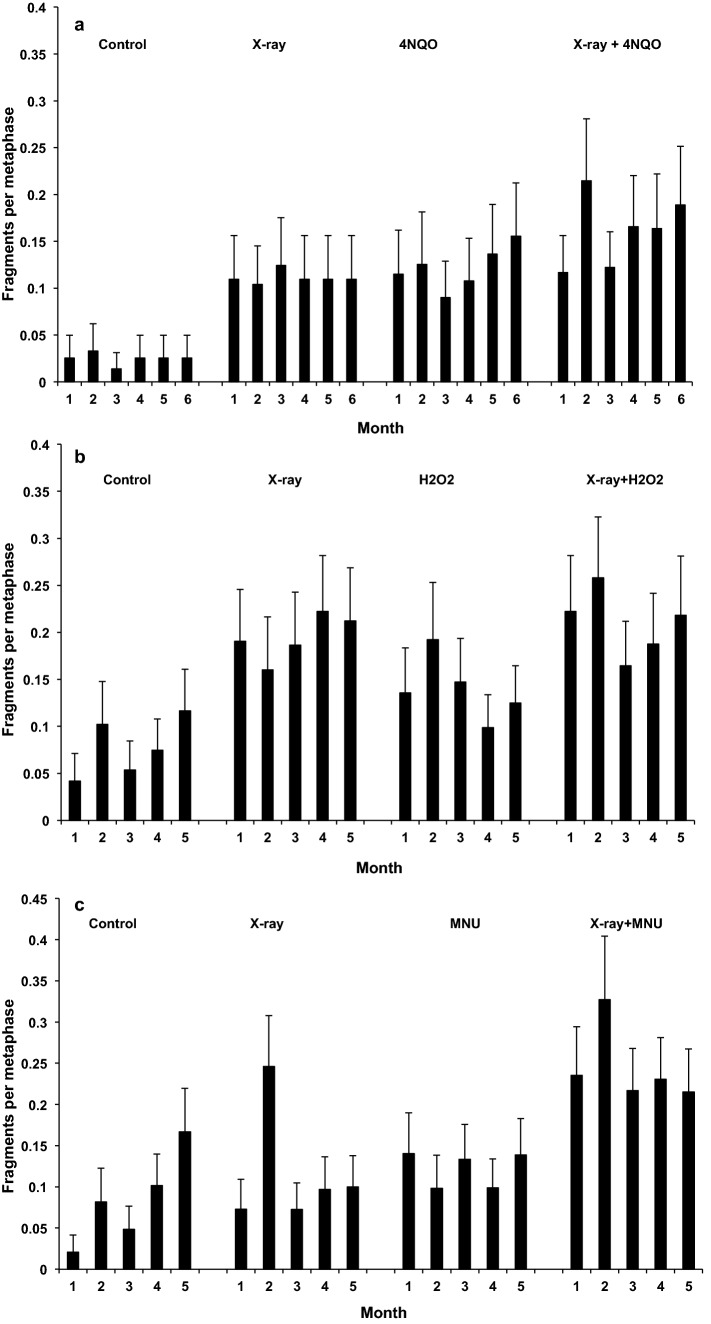
Acentric fragments per diploid metaphase after 1–6 months of chronic exposure to: 20 ng/ml 4NQO or sham-exposure and/or acute exposure to 1 Gy X-rays (**a**); 10 μM H_2_O_2_ or sham-exposure and/or acute exposure to 1 Gy X-rays (**b**); 0.25 μg/ml MNU or sham-exposure and/or acute exposure to 1 Gy X-rays (**c**). Error bars are Poisson errors.

Long-term exposure to 20 ng/ml 4NQO induced a few dicentric chromosomes (Fig. 5a; p = 0.004) and significant levels of acentric chromosome fragments (Fig. 6a; p < 0.001) above the levels found in sham-exposed cells after months 1–6 of exposure, with a significant difference in the responses for the different time points for fragments only (p = 0.038). Direct induction of dicentrics by 4NQO would be somewhat unexpected, as this is mechanistically difficult to explain and has not been reported previously. However, the dicentrics observed here are likely ‘derived’ ones. Such indirectly induced dicentrics are typically not accompanied by an acentric fragment. They are formed when unrepaired DNA lesions are converted into chromosomal aberrations during DNA replication.

Exposure to 1 Gy X-rays induced similar yields of dicentrics at all time points, irrespective of whether cells had been chronically exposed to 4NQO or not, with significant induction associated with exposure to X-rays only (p < 0.001) but with no significant interaction effect detected for the X-rays plus the chemical (p = 0.175). Pooling of all time points was therefore justified since no time dependent changes were observed (see Supplementary Table S1 online; p for time, all endpoints > 0.05). In total, 5 dicentrics and 11 acentrics were observed in 257 untreated cells, compared to 13 dicentrics and 57 acentrics in 300 4NQO-treated cells, whilst 310 cells exposed to 1 Gy X-rays contained 74 dicentrics/51 acentrics and 361 combined-treated cells contained 108 dicentrics/91 acentrics. See Supplementary Table S1 for the full data set on chromosomal aberrations.

**Figure 7:**
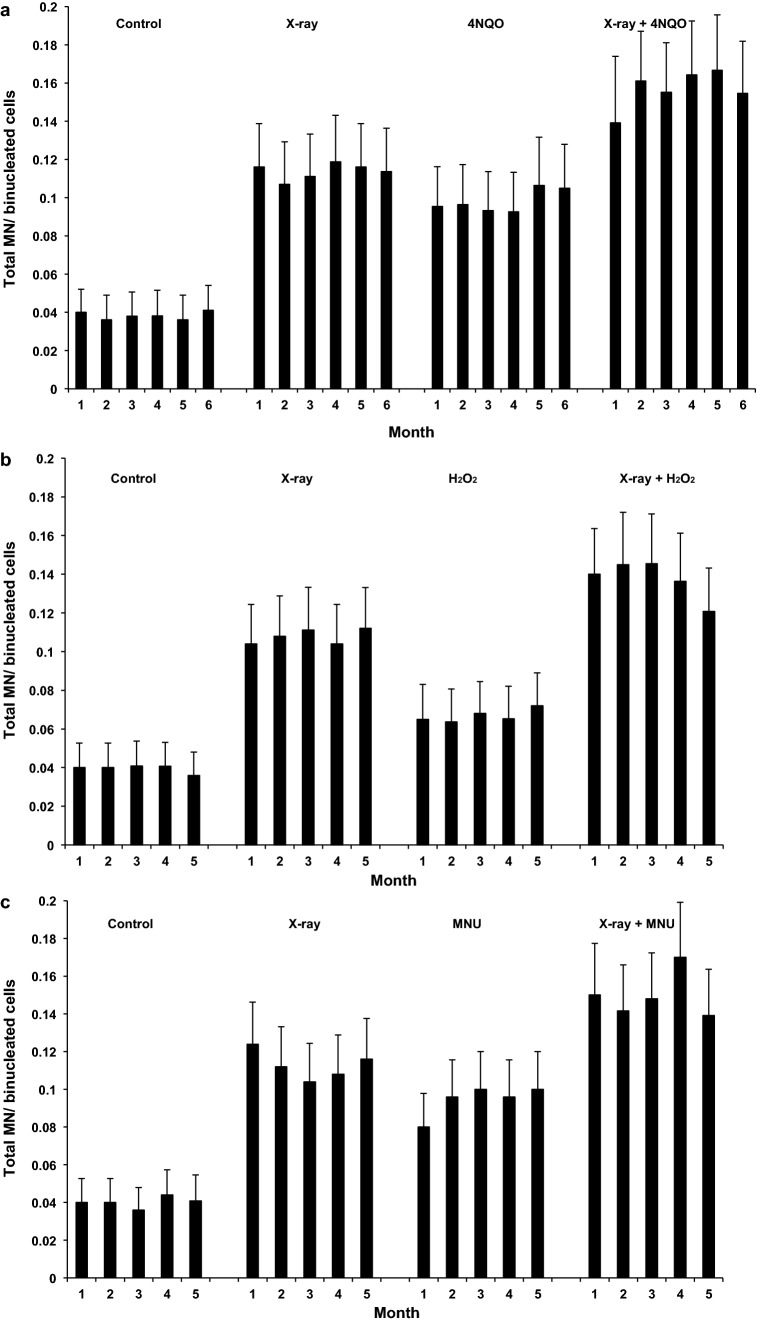
Micronuclei (MN) per binucleated cell after 1–6 months of chronic exposure to 20 ng/ml 4NQO or sham-exposure and/or acute exposure to 1 Gy X-rays (**a**), 10 μM H_2_O_2_ or sham-exposure and/or acute exposure to 1 Gy X-rays (**b**), 0.25 μg/ml MNU or sham-exposure and/or acute exposure to 1 Gy X-rays (**c**). Between 115 and 275 binucleated cells were scored per data point for (**a**), between 200 and 270 cells for (**b**) and between 200 and 250 cells for (**c**). Error bars are Poisson errors.

The yields of chromosome damage did not change significantly for any of the treatment groups during long-term exposure. Exposure to 20 ng/ml 4NQO increased micronuclei levels consistently above the baseline levels and in combination with X-rays induced a significant increase in micronuclei compared with X-rays alone (Fig. 7a and Supplementary Table S2; p < 0.001). This was consistent with an additive effect. As observed for chromosome aberrations, micronuclei formation did not change over the duration of exposure (p = 0.560) so that individual counts were pooled to give 54 micronuclei in 1407 binucleated untreated cells; 149 in 1310 X-irradiated cells; 122 in 1248 4NQO-treated cells and 189 in 1195 combined-treated cells.

Long-term exposure to 10 μM H_2_O_2_ did not induce any dicentric chromosomes (Fig. 5b) but a similar number of chromosome fragments as 1 Gy of X-rays (Fig. 6b) above the level found in sham-exposed cells after long-term exposure (p < 0.05). Exposure to 1 Gy X-rays induced a similar level of dicentrics at all time points, irrespective of whether cells had been chronically exposed to hydrogen peroxide or not. Chromosome fragment data show a slight, but not significant, increase in combined treatment samples compared to single treatments, consistent with an additive effect.

Exposure to 10 μM H_2_O_2_ increased micronuclei levels slightly, but consistently above the baseline levels and in combination with X-rays induced a significant increase in micronuclei compared with X-rays alone (Fig. 7b and Supplementary Table S2; p < 0.001). This was consistent with an additive effect. Micronuclei formation did not change over the duration of exposure so that individual counts were pooled to give 50 micronuclei in 1265 binucleated untreated cells; 132 in 1225 X-irradiated cells; 77 in 1150 H_2_O_2_-treated cells and 155 in 1130 combined X-rays and H_2_O_2_-treated cells.

Figure 5c shows that exposure to 0.25 μg/ml MNU did not induce a significant number of dicentrics but levels of chromosome fragments shown in Fig. 6c were similar to the samples treated with 1 Gy of X-rays and sham-exposed cells after 5 months of exposure. Exposure to 1 Gy X-rays induced a similar level of dicentrics at all time points, but levels tended to be slightly higher for cells that had been chronically exposed to MNU. This trend was, however, not significant. Chromosome fragment data show an increase in combined treatment samples compared to single treatments, consistent with an additive effect.

Figure 7c shows that exposure to 0.25 μg/ml MNU increased somewhat the micronuclei levels and in combination with X-rays induced a significant increase in micronuclei compared with X-rays alone (for all data set see Supplementary Table S2; p < 0.001). Individual counts for each treatment group were pooled because micronuclei formation did not change over the 5 months duration of exposure. 49 micronuclei were observed in 1220 binucleated untreated cells; 141 in 1250 X-irradiated cells; 118 in 1250 MNU-treated cells and 167 in 1120 combined-treated cells.

Our in vitro studies using human EBV-transformed lymphoblastoid cells have aimed to simulate exposure situations in which an individual is exposed chronically to a chemical genotoxicant and is then exposed to ionising radiation in a medical or accidental context.

In conclusion no significant acute effects on cell proliferation from any of the three agents was observed at the concentrations selected for chronic exposures, however, there was induction of various types of chromosomal damage such as micronuclei, dicentrics, and fragments—manifestations of chromosome injury which also are typical for radiation exposure. The crucial question is whether chronic exposure to these chemicals sensitises cells to subsequent radiation exposure or whether effects from either are just additive.

A similar study has been performed on sodium arsenite, which is a well-established carcinogen and genotoxicant using the same experimental protocol and the same model^25^. Here other genotoxic agents have been investigated, results of these studies are as summarised above.

The consistent finding of additive effects for the studied combined exposures suggests that, typically, radiation responses assessed with cytogenetic end points do not seem to be altered by long-term low level chemical exposure of GM1899A cells. Specifically, no significant adaptive responses or sensitising effects were observed in this cell line. Instead, the cellular response mechanisms for radiation damage, DNA repair, cell cycle checkpoint control and cellular survival/death pathways, seem to operate without any modulatory effects from chronic low-level chemical exposure in the systems analysed here. The obvious limitation of this study is the in vitro nature of the work which involved the use of an established human EBV-transformed lymphoblastoid cell line rather than primary human tissues. Therefore, this study is in itself insufficient to provide conclusive evidence on human health implications from combined exposures. The above conclusions are only valid for ionising radiation as a challenging agent and may depend on the use of this particular cell line*. *In vivo*,* supracellular and systemic aspects like inflammatory and immune responses need to be taken into account in future work. Although, the cytogenetic endpoints used here are currently the most suitable biomarkers for cancer risk, they are probably not informative for non-cancer effects like cardiovascular diseases which have more recently emerged as important medical conditions associated with exposure to low or moderate levels of a range of environmental hazards, including chemicals and ionising radiation^54^. Integration of sublethal concentrations and endpoints into risk assessment frameworks is important. This study does not deal with chronic toxicity effects arising at individual or population levels following long-term continuous or fluctuating exposure to chemicals at sublethal concentrations (not high enough to cause mortality or directly observable impairment following acute short-term exposure).

There are many examples of studies on combined exposures to ionising radiation and genotoxic agents including the improvement of tumour therapy by concurrent treatment with a chemical. However, the high doses and deterministic effects involved in these combined therapies cannot be simply related to low level combined effects.

We conclude, in agreement with the UNSCEAR 2000 Report^2^ that exposures of GM1899A cells to radiation and low level chemicals yield additive effects.

## Materials and methods

Most of the procedures described here were the same as in Nuta et al.^25^.

### Cells

GM 1899A, a normal human lymphoblastoid line (received from Dr M O’Donovan, Astra Charnwood, Loughborough, UK) was used for all experiments. Many studies on chemical exposures are performed using lymphoblastoid cells lines such as TK6, AHH-1^8,39,40^. Usage of lymphoblastoid cell lines established by in vitro infection with Epstein Barr Virus (EBV) as a reliable model system for carcinogen sensitivity, DNA damage/repair and other analyses has been consistent throughout the last decade. Since this is a study in which acute and long term (6 months) toxicity of the genotoxins were tested there was a reason to believe that the GM1899A cell line that was readily available in our lab is in close resemblance with the parent lymphocytes. Cells were grown in suspension at 37 °C in a humidified atmosphere of 95% air: 5% CO_2_ in Dutch Modified RPMI 1640 medium supplemented with 20% heat-inactivated fetal bovine serum, 2 mM sodium pyruvate, 2 mM l-glutamine and antibiotic/antimycotic (penicilin streptomycin solution)(Gibco Life Technologies, UK). For all experiments, asynchronous suspension cultures in the exponential phase of growth were seeded from the same batch and grown in T75 flasks.

### Treatment with chemicals

*N*-nitroso-*N*-methylurea (MNU) and 4-nitroquinoline-1-oxide (4NQO) were initially dissolved in DMSO and then further diluted in water. Hydrogen peroxide (H_2_O_2_) was diluted in water. Chemicals were supplied by Sigma-Aldrich, UK. DMSO concentrations were never higher than 0.1% for acute exposures and below 0.01% for chronic exposures. For acute exposures, cells seeded at 0.6 × 10^5^ cells/ml were treated with different concentrations of chemicals for 24 h. For long-term exposures, cells were cultured in the presence of 0 or 20 ng/ml 4NQO, 0 or 0.25 μg/ml MNU, and 0 or 10 μM H_2_O_2_. Cells were counted, split and medium and chemical renewed every 3.5 days.

### Irradiation

Every four weeks, aliquots of long-term chemical-treated cells were exposed at room temperature to 0 or 1 Gy of 250 kVp X-rays, with 11 mA and Al/Cu filtration, at a dose rate of approximately 1 Gy/min. The non-chemical exposed parallel controls were also X-rayed and sham X-rayed. Physical dosimetry was carried out with a calibrated Farmer dosemeter in the same geometry as the specimens.

### Cytokinesis-blocked micronucleus assay

Treated and sham-treated cells were cultured for 24 h in the presence of 3 μg/ml cytochalasin B (added 30 min post irradiation) and washed with medium. For the acute and the long-term exposures, cells were resuspended in 5 ml cold hypotonic solution (KCl 5.6 g/l), spun down again and fixed in 5 ml fixative (acetic-acid: methanol, 1:10 v/v) (Fisher Scientific, UK). Three drops of formaldehyde 37% (Polysciences, UK) were added and the fixative was changed twice after centrifugation at 600 rpm for 8 min. Fixed cell suspensions were dropped onto pre-cleaned microscope slides with a drawn-out Pasteur pipette and all slides were stained for 3.5–5 min in a 2% aqueous Giemsa solution (BDH Laboratory Supplies, UK), air-dried at room temperature and mounted in DPX mounting medium (Thermo Scientific, UK). Micronuclei in binucleated cells were scored by eye at 1000 × magnification with an Axioskop (ZEISS, Germany) microscope under oil immersion.

### Chromosomal aberration analyses

Colcemid at a concentration of 25 μg/ml was added to each cell culture 22 h after irradiation or mock-irradiation which was then returned to the incubator for 2 h. After this time cells were spun down, treated with prewarmed hypotonic KCl solution (5.6 g/l) and incubated for 15 min in a water bath at 37 °C, spun down and fixed three times in methanol: acetic acid (3:1 v/v). The slides were prepared and stained with Giemsa solution as described above. Scoring of dicentrics, acentric fragments and all other aberrations was carried out at 1,000 × magnification under oil immersion using the Metafer metaphase finding system (MetaSystems). All dicentrics were scored, noting whether or not they were accompanied by an acentric fragment. In addition, excess acentric fragments were scored, *i.e*. those not accompanied by a dicentric.

### Flow cytometric cell cycle analyses

Cells were harvested by centrifugation at 1200 rpm at 4 °C for 5 min and resuspended in PBS. 10^6^ cells were prepared per tube, fixed in cold ethanol (70%) and stained with 1 μg/ml propidium iodide solution. Cells were analysed in a FACSCalibur flow cytometer (BD Biosciences) with excitation at 488 nm.

### MTT assay

The CellTiter-Blue cell viability assay (Promega) was used. Triplicate wells containing cells treated with MNU at different concentrations were set in parallel with no-cell control wells which served as negative control to determine background fluorescence and with untreated cells control wells. Briefly, 96-well plates containing cells in culture medium were prepared and the test compound and vehicle controls were set up according to the protocol. Cells were cultured for the desired test exposure period, removed from the incubator and 20 µl/well of CellTiter-Blue reagent was added to each plate. After an incubation step, fluorescence at 560/590 nm was recorded using a plate-reading fluorometer. Calculation of results was executed according to the suppliers’ protocol.

### Data analyses

The “Dose Estimate” software was used to calculate Poisson errors^55^.

A subset of the control and treated data for each analysis and endpoint were tested for normality using the Anderson Darling normality testing. General linear model analysis of variance (ANOVA) was then carried out to investigate the effects of the experimental factors on the outcomes. For the pilot studies, for acute genotoxic effects of chemicals, the ANOVA model factor was chemical concentration and the response was percentage of binucleated cells or yield of micronuclei. For the medium term exposures to chemicals, the model factors were culture time and chemical for the response relative cell number, and concentration and time for the response percentage viability. For the main study, ANOVA was used to assess the impact of factors days in culture (0–160) with or without the chemicals on cell count. To investigate the role of radiation with or without chemicals on the observed number of dicentrics per metaphase, fragments per metaphase and total micronuclei per binucleated cell, the ANOVA factors were exposure month (1–6), radiation dose (0 or 1 Gy), and chemical (0 or concentrations as detailed in the main text). In each case, the effect of the irradiation and chemicals was also tested for evidence of interaction between these factors.

## Supplementary Information

Supplementary Information.

## Data Availability

All data generated and analysed during this study are included in this published article (and its Supplementary Information files).
