# Supplementary material for: Investigating the impact of long term exposure to chemical agents on the chromosomal radiosensitivity using human lymphoblastoid GM1899A cells
Source: Sci Rep. 2021 Jun 16;11:12616. doi: 10.1038/s41598-021-91957-y (PMC8209142; doi:10.1038/s41598-021-91957-y)
Supplement: Supplementary file 1 — Supplementary Information. [file 41598_2021_91957_MOESM1_ESM.pdf]

Impact of long term exposure to chemical agents on the chromosomal radiosensitivity of human lymphoblastoid cells

Otilia Nuta, Simon Bouffler, David Lloyd, Liz Ainsbury, Ovnair Sepai, Kai Rothkamm

Supplementary Table S1 (a): Chromosomal aberration data for 20 ng / ml 4NQO long-term exposure experiment.

Treatment	Month	Aberrations					Number of metaphases scored
		Dicentrics	Fragments	Chromatid breaks	Minutes	Other aberrations	
Control	1	1	2	0	8	0	43
	2	0	2	0	6	0	39
	3	1	1	0	7	0	46
	4	1	2	0	8	0	43
	5	1	2	0	8	0	43
	6	1	2	0	8	0	43
1Gy X-rays	1	12	8	2	5	0	50
	2	13	10	1	8	0	62
	3	13	9	2	7	0	48
	4	12	8	2	5	0	50
	5	12	8	2	5	0	50
	6	12	8	2	5	0	50
20 ng / ml 4NQO	1	3	10	0	0	1 exchange	52
	2	1	8	0	4	1 exchange	40
	3	2	8	0	3	2 exchanges	60
	4	2	9	0	4	2 exchanges	52
	5	2	10	0	3	3 exchanges, 2 gaps	48
	6	3	12	0	4	2 exchanges	48
20 ng / ml 4NQO + 1Gy X-rays	1	18	14	0	3	1gap, 1triradial	76
	2	20	18	1	6	3 gaps	49
	3	18	17	2	4	2 gaps	84
	4	18	15	2	0	1 gap, 1 exchange	56
	5	18	13	1	0	2 gaps, 2 exchanges	48
	6	16	14	1	0	1 gap, 1 exchange	48

Supplementary Table S1 (b): Chromosomal aberration data for 10 μM H_2O_2 long-term exposure experiment.

Treatment	Month	Aberrations					Number of metaphases scored
		Dicentrics	Fragments	Chromatid breaks	Minutes	Other aberrations	
Control	1	1	2	0	3	0	48
	2	0	5	0	2	0	49
	3	0	3	0	3	0	56
	4	0	5	0	4	0	67
	5	0	7	0	4	0	60
1Gy X-rays	1	13	12	4	2	0	63
	2	14	8	4	2	0	50
	3	12	11	5	2	0	59
	4	13	14	3	3	0	63
	5	16	14	3	3	0	66
10 μM H_2O_2	1	0	8	3	1	0	59
	2	0	10	3	4	0	52
	3	0	10	3	2	0	68
	4	0	8	1	4	0	81
	5	0	10	1	4	0	80
10 μM H_2O_2 + 1Gy X-rays	1	14	14	3	6	0	63
	2	15	16	3	3	0	62
	3	15	12	4	6	0	73
	4	15	12	4	6	0	64
	5	14	12	4	6	0	55

Supplementary Table S1 (c): Chromosomal aberration data for 0.25 µg/ml MNU long-term exposure experiment.

Treatment	Month	Aberrations					Number of metaphases scored
		Dicentrics	Fragments	Chromatid breaks	Minutes	Other aberrations	
Control	1	1	1	0	10	0	48
	2	0	4	0	8	0	49
	3	1	3	0	12	0	62
	4	1	7	0	0	0	69
	5	0	10	0	0	0	60
1Gy X-rays	1	12	4	3	8	0	55
	2	14	16	1	8	0	65
	3	13	5	4	5	0	69
	4	11	6	0	3	0	62
	5	12	7	0	3	0	70
0.25 µg/ml MNU	1	2	8	0	0	3 exchanges	57
	2	0	6	1	5	0	61
	3	3	10	0	0	0	75
	4	1	8	0	0	0	81
	5	1	10	0	0	0	72
0.25 µg/ml MNU + 1Gy X-rays	1	16	16	0	2	3 gaps, 2 exchanges	68
	2	17	18	0	4	3 gaps	55
	3	16	18	0	3	4 gaps, 1 exchange	83
	4	16	21	0	0	0	91
	5	14	17	0	0	0	79

Supplementary Table S2 (a): Micronucleus assay data for 20 ng / ml 4NQO long-term exposure experiment.

Treatment	Month	Number of BN cells scored	BN cells with 0 MN	BN cells with 1 MN	BN cells with 2 MN	BN cells with 3 MN	Total MN
Control	1	275	264	11	0	0	11
	2	221	213	8	0	0	8
	3	237	228	9	0	0	9
	4	210	202	8	0	0	8
	5	221	213	8	0	0	8
	6	243	233	10	0	0	10
1Gy X-rays	1	224	200	22	2	0	26
	2	215	194	19	2	0	23
	3	225	202	21	2	0	25
	4	202	180	20	2	0	24
	5	224	200	22	2	0	26
	6	220	196	23	1	0	25
20 ng / ml 4NQO	1	220	200	19	1	0	21
	2	218	200	15	3	0	21
	3	225	205	17	2	0	21
	4	216	200	12	4	0	20
	5	169	153	14	2	0	18
	6	200	181	17	2	0	21
1Gy X-rays+ 20 ng / ml 4NQO	1	115	100	14	1	0	16
	2	236	200	34	2	0	38
	3	232	202	25	4	1	36
	4	207	175	30	2	0	34
	5	198	169	26	2	1	33
	6	207	179	25	2	1	32

Supplementary Table S2 (b): Micronucleus assay data for 10 µM H_2O_2 long-term exposure experiment.

Treatment	Month	Number of BN cells scored	BN cells with 0 MN	BN cells with 1 MN	BN cells with 2 MN	BN cells with 3 MN	Total MN
Control	1	250	240	10	0	0	10
	2	250	240	10	0	0	10
	3	245	235	10	0	0	10
	4	270	259	11	0	0	11
	5	250	241	9	0	0	9
1Gy X-rays	1	250	225	24	1	0	26
	2	250	225	23	2	0	27
	3	225	202	21	2	0	25
	4	250	226	22	2	0	26
	5	250	225	22	3	0	28
10 µM H_2O_2	1	200	187	13	0	0	13
	2	220	206	14	0	0	14
	3	250	234	15	1	0	17
	4	230	215	15	0	0	15
	5	250	234	14	2	0	18
1Gy X-rays+ 10 µM H_2O_2	1	250	220	26	3	1	35
	2	200	174	24	1	1	29
	3	220	192	25	2	1	32
	4	220	193	24	3	0	30
	5	240	213	25	2	0	29

Supplementary Table S2 (c): Micronucleus assay data for 0.25 µg/ml MNU long-term exposure experiment.

Treatment	Month	Number of BN cells scored	BN cells with 0 MN	BN cells with 1 MN	BN cells with 2 MN	BN cells with 3 MN	Total MN
Control	1	250	240	10	0	0	10
	2	250	240	10	0	0	10
	3	250	241	9	0	0	9
	4	250	239	11	0	0	11
	5	220	211	9	0	0	9
1Gy X-rays	1	250	222	25	3	0	31
	2	250	224	24	2	0	28
	3	250	225	24	1	0	26
	4	250	225	23	2	0	27
	5	250	223	25	2	0	29
0.25 µg/ml MNU	1	250	231	18	1	0	20
	2	250	227	22	1	0	24
	3	250	227	21	2	0	25
	4	250	228	20	2	0	24
	5	250	227	21	2	0	25
1Gy X-rays+ 0.25 µg/ml MNU	1	200	171	28	1	0	30
	2	240	208	30	2	0	34
	3	250	219	26	4	1	37
	4	200	168	30	2	0	34
	5	230	202	25	2	1	32
